# Mental Health of University Students When Returning to Face-to-Face Classes: A Cross-Sectional Study

**DOI:** 10.3390/bs13060438

**Published:** 2023-05-23

**Authors:** Edwin Gustavo Estrada-Araoz, Judith Annie Bautista Quispe, Lizbeth Maribel Córdova-Rojas, Euclides Ticona Chayña, Humberto Mamani Coaquira, Jhony Huaman Tomanguilla

**Affiliations:** 1 Facultad de Educación, Universidad Nacional Amazónica de Madre de Dios, Puerto Maldonado 17001, Peru; 2 Facultad de Ciencias Naturales y Aplicadas, Universidad Nacional Intercultural Fabiola Salazar Leguía de Bagua, Bagua 01721, Peru; jbautistaq@unibagua.edu.pe (J.A.B.Q.); mcordova@unibagua.edu.pe (L.M.C.-R.); eticonac@unibagua.edu.pe (E.T.C.); jhuaman@unibagua.edu.pe (J.H.T.); 3 Facultad de Ciencias de la Educación, Escuela Profesional de Educación Primaria, Universidad Nacional del Altiplano, Puno 21001, Peru; hmamanic@unap.edu.pe

**Keywords:** mental health, depression, anxiety, stress, face-to-face classes, post-pandemic, students, COVID-19

## Abstract

Depression, anxiety and stress are multifactorial affective disorders that could manifest through a set of symptoms, both physical and psychological, that affect the quality of life and performance of people who suffer from them. In this sense, the present research had the objective of evaluating depression, anxiety and stress in students of the Faculty of Engineering of a Peruvian public university when returning to face-to-face classes. The research was developed under a quantitative approach and is of a non-experimental design of the descriptive cross-sectional type. The sample consisted of 244 students who responded to the Depression, Anxiety and Stress Scale, an instrument with adequate psychometric properties. According to the results, the students presented low levels of depression and anxiety. However, they showed moderate levels of stress. On the other hand, it was found out that the three variables were directly and significantly related. In the same way, it was found that there were statistically significant differences regarding the levels of depression, anxiety and stress related to gender, age group, family responsibilities and professional career. Finally, it was concluded that there were symptoms of depression, anxiety and stress in students of the Faculty of Engineering of a Peruvian public university when returning to face-to-face classes.

## 1. Introduction

Since 2020, the COVID-19 pandemic has caused great changes in people’s lifestyles, mainly due to its health and social consequences. In the educational field, the teaching–learning modality was modified, which changed from face-to-face to virtual [1]. However, from the first two months of 2022, the number of infections and deaths associated with COVID-19 decreased significantly worldwide due to vaccination campaigns [2]. In Peru, many activities that were carried out virtually during the pandemic were carried out again in person. In this way, starting from April 2022, students gradually returned to face-to-face classes after complying with biosafety protocols [3].

The return to university classrooms and its implicit adaptive process after two years of virtuality and mandatory isolation could be affecting the emotional state of students [4]. It is regularly argued that the professional training process is associated with a series of stressors, among which stand out the multiple demands generated by tasks, the short time to develop them (academic overload), evaluations and the number of hours per day of teaching [5]. This could be causing the wear and tear of emotional resources and causing some conditions that disrupt mental health.

Mental health is defined as a state of well-being in which each person realizes his or her own potential and can cope with the normal stresses of life, develop her or himself productively and fruitfully, and be able to contribute to him or herself and the community [6]. However, the admission and attendance of students at the university could affect their emotional state, since this formative period is considered critical and very stressful as they leave their homes, have to adapt to a new social environment, must face greater academic pressure as previously described and have more access to the consumption of alcoholic beverages and psychoactive substances [7].

Actually, according to the data collected in 21 countries as part of the World Mental Health Survey prepared by the World Health Organization (WHO), one-fifth of higher education students in all countries had various mental disorders, especially mood disorders [8]. These disorders that affect the emotional state of students are related to three variables on which this research will focus: depression, anxiety, and stress (DAS).

Depression involves a wide range of emotional problems, from sadness to a pathological suicidal state. It was conceptualized as an affective disorder that is evidenced through manifestations of frustration, sadness, and changes in people’s mood [9]. In cases in which students are not properly treated, they could have a negative impact on relationships with peers or family and affect academic performance. Likewise, there is also a danger that students acquire habits, such as the consumption of alcohol or psychoactive substances, to try to overcome the disorder. On the other hand, major depressive disorder has been found to be one of the main causes of suicidal behavior in young people [10].

Regarding anxiety, it constitutes an emotional response that has an activating and facilitating function and, at the same time, plays an adaptive role in situations that are perceived as threatening [11]. In addition, anxious students suffer from learning difficulties and an inability to solve problems. Psychological and physical symptoms include chills on the hands and lips, dry mouth, frequent urination and sleep disturbances [12]. It is necessary to specify that occasional anxiety is common, but not when it comes to intense, persistent and excessive fears and worries that could be exacerbated and cause anxiety disorder.

With regard to stress, it is a behavior or response of the body to a variety of external and internal pressures, an adaptive and emergency process being important for the survival of the human being [13]. In any case, stress is a consequence of the relationship between individuals and their environment [14]. As a matter of fact, stress is an automatic response of the organism to any event that is imposed and perceived as threatening [15], causing the nervous system to be stimulated and react by producing changes to psychological (mental) or physiological (physical) levels that occur in a particular way between a person and his situation [16]. Therefore, in order to control the experience of stress, a person could try to alter their environment or learn forms to modify his way of reacting to a specific situation, and it is important to try to get the person and his environment to a point of adaptation [17].

There are investigations that evaluated for the presence of DAS in university students; however, they were developed in the context of a pandemic. In Cuba, research was carried out to evaluate the mental health of Stomatology students and concluded that the levels of DAS were severe [18]. In Peru, researchers also inquired about the presence of DAS and determined that the students presented various disorders such as depression, anxiety, and somatization [19]. In Bangladesh, DAS was evaluated among students at Jahangirnagar University and it was found that there was a low prevalence of the mentioned mental conditions [20].

Currently, no research has yet been reported on DAS in Peruvian students and, specifically, in university students during the return to face-to-face classes after the COVID-19 pandemic. This is despite the fact that, as previously seen, DAS has implications for student performance, personal well-being and quality of life. In this sense, the present research work becomes relevant due to its originality. Based on its findings, it is expected that the university authorities could manage the presence of professionals in order to prevent and detect problems with their emotional state and, on the other hand, promote the development of various psychoeducational strategies and protective factors allowing them to face the various difficulties of higher education.

Most importantly, the objective of this research was to evaluate depression, anxiety and stress in students of the Faculty of Engineering of a Peruvian public university when returning to face-to-face classes.

## 2. Materials and Methods

### 2.1. Design

The research approach was quantitative since it was focused on numerical measurement as well as the use of statistics to determine the behavior patterns of the participants. Regarding the design, it was non-experimental since the DAS variables were not deliberately manipulated; they were only observed. Regarding the type of design, it was descriptive cross-sectional as the analysis of the characteristics of the variables was developed and the data collection was carried out in a single moment [21].

### 2.2. Participants

The population was composed of 670 students enrolled in the 2022-II cycle at the Faculty of Engineering of a public university in Puerto Maldonado, Peru. Regarding the sample, it was integrated by 244 students, a number determined by probabilistic sampling with a confidence level of 95% and a significance of 5%. Table 1 shows that 71.7% of the participants were male and 28.3% were female. Regarding the age group, 82% were between 16 and 25 and 18% were older than 25 years old. Likewise, 74.2% indicated that they did not have a family responsibility, while 25.8% did have one. On the other hand, 46.3% of students belonged to the professional career of Systems Engineering and Informatics, 22.1% were from Forestry and Environmental Engineering, 18.9% from Agro-industrial Engineering and 12.7% from Veterinary Medicine and Zootechnics.

### 2.3. Instruments

A survey was performed for data collection, which was structured in two sections. In the first section, participants were asked for sociodemographic information (gender, age group, and professional career). In the second section, the Depression, Anxiety and Stress Scale (DASS-21) was applied, which was created by Lovibond et al. [22] and adapted to the Peruvian reality by Corrales et al. [18]. This scale is made up of 21 items quantitatively rated using a 4-point Likert scale that ranges from 0 (it has not happened to me) to 3 (it has happened to me a lot) and are distributed in 3 dimensions: depression (items from 1 to 7), anxiety (items from 8 to 14) and stress (items from 15 to 21). Its psychometric properties were determined in a previous study through the content validity and reliability process. In this sense, it was established that the scale had an adequate content validity (Aiken’s V = 0.801) and reliability level (α = 0.838).

### 2.4. Procedure

To carry out the data collection, the respective permits were obtained by the university authorities. Subsequently, once they met in person with the students, the purpose of the research was explained to them and the respective orientations were given, and the scale was developed. This procedure lasted approximately 15 min. Finally, a database was created with the responses of students.

### 2.5. Data Analysis

Data analysis was performed at a descriptive and inferential level. The descriptive analysis was developed through the use of a figure and three tables that were obtained by the SPSS V.25 Software. Afterward, a Pearson correlation analysis was performed in order to find out if the study variables were significantly related. Likewise, the Student’s *t*-test was used to find out if there were statistically significant differences with respect to the DAS according to sex, age group and family responsibility. Finally, the Chi-Square test was used to find out if professional careers were significantly associated with DAS.

### 2.6. Ethical Aspects

Regarding the ethical aspects, the research had the endorsement of the Institutional Ethics Committee. Likewise, it should be specified that the students were informed about the purpose and nature of the research and provided their informed consent, guaranteeing the anonymous and voluntary nature of their participation at all times.

## 3. Results

**Figure 1:**
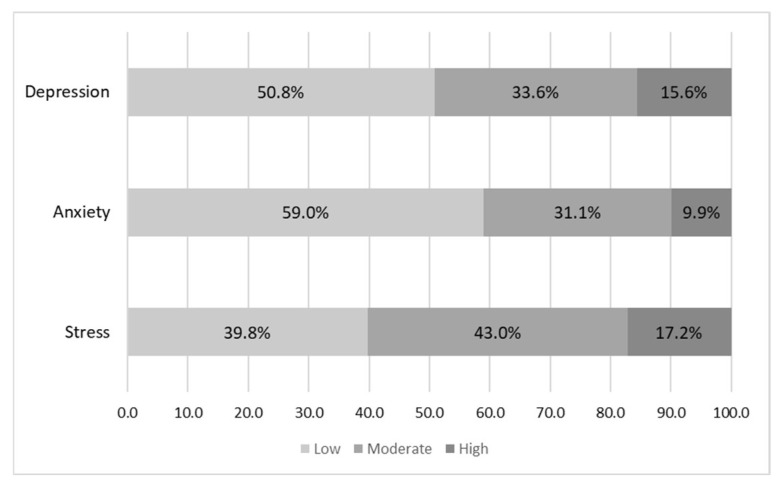
Levels of depression, anxiety and stress.

Figure 1 shows that the predominant level of depression was low (50.8%), followed by the moderate level (33.6%) and the high level (15.6%). Regarding anxiety, the low level also predominated (59%), followed by the moderate level (31.1%) and the high level (9.9%). Regarding stress, the moderate level (43%) predominated, followed by the low level (39.8%) and the high level (17.2%). The data provided indicate that students presented certain emotional, cognitive and physiological reactions that affected their health and were possibly caused by the high academic demands, the demands of face-to-face university education and the process of adaptation to the new normality after more than two years of virtual classes.

Table 2 shows that the main symptoms associated with depression were feeling discouraged and sad, having little initiative to do things, not feeling any positive emotion and feeling that there was nothing that make them get their hopes up.

Table 3 shows that the most recurrent symptoms associated with anxiety were being worried about situations in which they might panic and make a fool of themselves, noticing a dry feeling in their mouth, feeling that they were on the verge of panic and feeling scared for no relevant reason.

In Table 4, it can be observed that the main symptoms associated with stress were having difficulty relaxing, feeling that they were expending a large amount of energy, having difficulty releasing tension and feeling angry easily.

Table 5 shows the results resulting from the correlation analysis between the studied variables. In this sense, there is a direct and significant relationship between depression and anxiety (r = 0.832); between depression and stress (r = 0.847); and between anxiety and stress (r = 0.852).

When comparing the DAS according to gender, there are statistically significant differences between men and women (Table 6). In fact, this result suggests that women are more likely than men to experience higher levels of DAS.

In Table 7, the DAS was compared according to the age group and it was determined that there are statistically significant differences between the students who are between 16 and 25 years old and the students older than 26 years old. In this sense, this result indicates that younger students are more likely to experience higher levels of DAS than adult students.

In Table 8, the DAS was compared related to family responsibility of students and it was determined that there are statistically significant differences between students who have a family responsibility and those who do not. The obtained result indicates that the family responsibility can have a negative impact on the mental health of students, that is, it would cause an increase in the levels of DAS.

According to Table 9, students of Forest Engineering and Environment presented slightly higher levels of depression than students of other professional careers. However, when analyzing the association through the Chi-Square test, it was determined that it was not statistically significant.

As we can see in Table 10, students of Forest Engineering and Environment, as well as those of Veterinary Medicine and Zootechnics, presented slightly higher levels of anxiety than students of other professional careers. When analyzing the mentioned relation through the Chi-Square test, it was statistically significant.

As can be seen in Table 11, the students of Forestry and Environmental Engineering, as well as those of Veterinary Medicine and Zootechnics, presented slightly higher levels of stress than the students of the other professional careers. When analyzing the mentioned relation through the Chi-Square test, it was also determined that it was statistically significant.

## 4. Discussion

In recent decades, research aimed at studying the mental health of university students has significantly increased since psychosocial variables are considered determining factors in emotional state and personal well-being necessary for adequate academic and social development. The return to face-to-face classes that arose after the health emergency caused by COVID-19 could have caused some mental conditions, such as DAS, due to the demands that characterize university education. For this reason, in the present investigation, the DAS was evaluated in students of the Faculty of Engineering of a Peruvian public university when returning to face-to-face classes.

It was found that the students were characterized by low levels of depression; that is, they infrequently reported feelings of sadness and hopelessness, which could be explained because the health emergency caused by COVID-19 has been controlled and also by the low probability of being infected, hospitalized or dying. However, there is also a considerable percentage of students reporting moderate levels of depression, a situation characterized by changes in mood and frustration possibly associated with the difficulties caused by readaptation to face-to-face classes and the natural complexity of attending classes at the university. This result converges with what was reported in Argentina, where they found that university students had low levels of depression in the context of face-to-face education after the pandemic [23].

In the same way, it was found that the predominant level of anxiety was also low, which means that the majority of students hardly showed behaviors or manifestations of worry or physical, mental or emotional agitation in the face of complex situations that imply instability or uncertainty. Although it seems an encouraging finding, it should be considered that almost a third of all students showed moderate levels of anxiety, characterized mainly by showing worries about the development of their academic responsibilities and feeling that they are on the verge of panic, a situation where their heart rate sped up. In this regard, it should be noted that the level of anxiety was lower than that found in a study carried out in Bangladesh where the authors analyzed the presence of DAS among university students and determined that more than 40% of them had extremely severe anxiety [20].

On the other hand, it was found that the predominant level of stress was moderate, characterized mainly by the presence of cognitive, emotional and physiological reactions that affected the emotional state of the students. This could have been caused by the continuous academic demands of university education and the process of adaptation to face-to-face education after more than two years of virtual education. Similar results were obtained in Bolivia, where researchers evaluated the emotional situation of university students at the end of the pandemic and found that the prevailing stress level was moderate [24]. However, the results found are lower than those reported in Cuba, where research was carried out to evaluate the mental health of Stomatology students and determined that the majority suffered from high levels of stress [18].

Mental conditions related to emotional state, such as DAS, frequently appear during youth, a period that coincides with attendance in classes at the university. However, students rarely receive support to overcome these conditions. These problems are related to a higher incidence of physical and emotional problems in the medium and long term, marginalization from the labor market, sleep disorders and dysfunctional relationships, among other problems [25]. For this reason, the need arises to promote detection campaigns and timely treatment that allow students to overcome the various stressors they face during their professional training and in daily life.

An interesting finding indicates that depression was directly and significantly related to anxiety and stress. Likewise, anxiety was also directly and significantly related to stress. Similar results were obtained in Spain, where researchers evaluated the mental health of university students and also determined that the three variables were directly and significantly related [26].

Likewise, it was found that women presented slightly higher levels of DAS compared to men. This finding could be explained from two perspectives. On one hand, they tend to externalize emotional and physiological manifestations in stressful contexts. On the other hand, in addition to their academic responsibilities, they assume additional tasks at home, such as family responsibilities, childcare and other domestic activities [27]. In this regard, some research supports the findings found. In Brazil, it was determined that one of the factors associated with DAS was gender, that is, there was a higher prevalence in women than in men [28]. In addition, a study was carried out in Spain to find out the variables associated with DAS in university students and concluded that the female gender was one of them [29]. Similarly, in Bangladesh researchers also analyzed the association between DAS and sociodemographic variables and found that women were more likely to suffer from the previously mentioned mental health problems [20].

Another finding shows that the youngest students presented higher levels of DAE than adult students. This could be explained by the fact that many young students are adapting themselves to the transition from the regular basic educational system to university and its high academic demands. Likewise, adult students could have formally or informally developed some strategies to face the difficulties of university education, the main sources of DAE [30]. The described result was corroborated by some investigations, which also determined that age was a factor associated with the prevalence of DAE [31,32,33,34]. In fact, age is an important factor to consider when studying the prevalence of DAE and can be useful to identify population groups that may need particular attention in terms of prevention, diagnosis and treatment.

It was also found that students with family responsibilities had higher levels of DAE than students who did not. This is logical since, in addition to academic responsibilities, students have to work to meet the basic needs of their families, a situation that would make their situation more complex and make them more vulnerable to suffering from mental illnesses. This result coincides with an investigation carried out in Peru, where researchers found that having a family and working to cover expenses associated with their maintenance can have a significant impact on the level of DAE of students [35].

Finally, it was determined that studying some professional careers, such as Forest Engineering and Environment or Veterinary Medicine and Zootechnics, was associated with higher levels of anxiety and stress. This result may have an important implication for students considering careers in these areas, as well as for college, as they may need to implement additional strategies to help students handle and reduce stress and anxiety.

This research addresses topics associated with the emotional state of university students during the post-pandemic context, poorly studied but very relevant. However, it is necessary to specify some limitations. Firstly, the sample size is relatively small and it is also homogeneous, which implies caution when interpreting the results. Second, the findings are based entirely on data obtained from self-administered instruments, which could have led to subjective judgments by the participants. Anyway, it is expected that in future research, the size of the sample will be expanded to include students from other professional careers, and various sociocultural characteristics and data collection instruments will be used to complement those that were applied and give more objectivity to the aforementioned process.

## 5. Conclusions

Mental health problems are one of the main causes of disability and a major public health problem worldwide due to the difficulties in therapeutic management and the increase in their prevalence in recent years. In this regard, DAS has been considered as important indicators of mental health that, if they are not treated, can have a negative impact on people’s quality of life.

In the present investigation, it was concluded that students from the Faculty of Engineering of a public university in Peru had low levels of depression and anxiety, but presented moderate levels of stress upon returning to in-person classes. Likewise, it was identified that the main symptoms associated with DAS reported by the students were feeling down and sad, being worried about situations in which they could panic and make a fool of themselves and having difficulty relaxing. Similarly, it was found that the three variables were directly and significantly related. Finally, some sociodemographic and academic variables such as gender, age group, family responsibility and the professional career students were studying in were associated with the levels of DAS. These results are important because they are novel in the current post-pandemic period and allow for a better understanding of the implications of returning to in-person classes for the mental health of students.

Based on the above, it would be important for university authorities to develop and implement strategies to assess, prevent and promote mental health among students. Likewise, the results emphasize the need for students to learn and develop effective coping strategies to manage the stress and pressure associated with their careers. This is especially relevant since they constantly face a heavy workload and significant levels of stress, which can increase the risk of developing mental health problems. By doing so, the university can improve the quality of life of its students and promote their academic performance and personal well-being. Finally, future research should delve into the topic of student mental health and explore other relevant variables that may influence students’ well-being.

## Figures and Tables

**Table 1:** Socio-demographic characteristics in the study sample.

Socio-Demographic Characteristics		n = 244	%
Gender	Male	175	71.7
	Female	69	28.3
Age group	From 16 to 25 years old	200	82.0
	Older than 25 years old	44	18.0
Family responsibilities	Yes	63	25.8
	No	181	74.2
Professional careers	Agro-industrial Engineering	46	18.9
	Forest Engineering and Environment	54	22.1
	Systems and Computer Engineering	113	46.3
	Veterinary Medicine and Zootechnics	31	12.7

**Table 2:** Symptoms associated with depression.

Items	Min	Max	M	SD
I have not been able to feel any positive emotion.	0	3	1.24	0.821
It was difficult for me to have initiative to do things.	0	3	1.30	0.851
I felt that there was nothing that make me get my hopes up.	0	3	1.19	0.929
I have felt discouraged and sad.	0	3	1.43	0.834
I have been unable to get excited about anything.	0	3	1.18	0.833
I have felt that I was not worth much as a person.	0	3	1.02	0.938
I have felt that life has no meaning	0	3	1.01	0.914

**Table 3:** Symptoms associated with anxiety.

Items	Min	Max	M	SD
I have noticed a dry feeling in my mouth.	0	3	1.09	0.843
I had difficulty breathing.	0	3	0.86	0.722
I had tremors.	0	3	0.92	0.782
I have been worried about situations where I might panic and make a fool of myself.	0	3	1.32	0.905
I felt that I was on the verge of panic.	0	3	1.00	0.796
I have noticed alterations in my heart without making physical effort.	0	3	0.93	0.783
I’ve been feeling scared for no relevant reason.	0	3	0.95	0.748

**Table 4:** Symptoms associated with stress.

Items	Min	Max	M	SD
It took me a long time to release the tension.	0	3	1.38	0.915
I have tended to overreact to situations.	0	3	1.33	1.010
I have felt that I was expending a large amount of energy.	0	3	1.38	0.955
I have felt agitated.	0	3	1.04	0.776
I have found it difficult to relax.	0	3	1.43	0.956
I have not tolerated anything that prevented me from continuing with what I was doing.	0	3	1.11	0.881
I have tended to get angry easily.	0	3	1.33	1.010

**Table 5:** Correlation between depression, anxiety and stress.

Variables	Depression	Anxiety	Stress
Depression	1	-	-
Anxiety	0.832 ***	1	-
Stress	0.847 ***	0.852 ***	1

Note = *** *p* < 0.001.

**Table 6:** Comparison of the means of the variables of depression, anxiety and stress related to gender.

Variables	Male		Female		t
	M	SD	M	SD	
Depression	7.84	5.418	9.72	5.139	−2.482 *
Anxiety	6.19	5.248	9.32	5.103	−4.228 ***
Stress	8.28	5.492	10.88	4.972	−3.424 **

Note = * *p* < 0.05, ** *p* < 0.01, *** *p* < 0.001.

**Table 7:** Comparison of the means of the variables of depression, anxiety and stress related to age group.

Variables	From 16 to 25 Years Old		Older than 25 Years Old		t
	M	SD	M	SD	
Depression	8.98	5.459	5.64	4.166	3.817 ***
Anxiety	7.65	5.417	4.48	4.438	3.619 ***
Stress	9.51	5.367	6.77	5.417	3.058 **

Note = ** *p* < 0.01, *** *p* < 0.001.

**Table 8:** Comparison of the means of the variables of depression, anxiety and stress related to family responsibilities.

Variables	With Family Responsibilities		Without Family Responsibilities		t
	M	SD	M	SD	
Depression	9.63	5.219	7.93	5.403	2.171 *
Anxiety	8.35	5.436	6.63	5.312	2.200 *
Stress	10.62	5.464	8.46	5.372	2.737 **

Note = * *p* < 0.05, ** *p* < 0.01.

**Table 9:** Association between depression and professional careers.

Variable		Depression			X^2^
		Low	Moderate	High	
Professional careers	Agro-industrial Engineering.	22 (47.8%)	17 (37.0%)	7 (15.2%)	9.632
	Forest Engineering and Environment	21 (38.9%)	22 (40.7%)	11 (20.4%)	
	Systems and Computer Engineering	67 (59.3%)	34 (30.1%)	12 (10.6%)	
	Veterinary Medicine and Zootechnics	14 (45.2%)	9 (29.0%)	8 (25.8%)	

**Table 10:** Association between anxiety and professional careers.

Variable		Anxiety			X^2^
		Low	Moderate	High	
Professional careers	Agro-industrial Engineering.	31 (67.4%)	10 (21.7%)	5 (10.9%)	22.647 **
	Forest Engineering and Environment	23 (42.6%)	26 (48.1%)	5 (9.3%)	
	Systems and Computer Engineering	76 (67.3%)	31 (27.4%)	6 (5.3%)	
	Veterinary Medicine and Zootechnics	14 (45.2%)	9 (29.0%)	8 (25.8%)	

Note = ** *p* < 0.01.

**Table 11:** Association between stress and professional careers.

Variable		Stress			X^2^
		Low	Moderate	High	
Professional careers	Agro-industrial Engineering.	19 (41.3%)	18 (39.1%)	9 (19.6%)	16.399 *
	Forest Engineering and Environment	17 (31.5%)	28 (51.9%)	9 (16.7%)	
	Systems and Computer Engineering	50 (44.2%)	51 (45.1%)	12 (10.6%)	
	Veterinary Medicine and Zootechnics	11 (35.5%)	8 (25.8%)	12 (38.7%)	

Note = * *p* < 0.05.

## Data Availability

Not applicable.
